# Pathogenetic substantiation of depressive disorders in old age as factors of premature brain aging

**DOI:** 10.1192/j.eurpsy.2025.1372

**Published:** 2025-08-26

**Authors:** A. P. Sidenkova

**Affiliations:** 1 Psychiatry, Ural State Medical University, Ekaterinburg, Russian Federation

## Abstract

**Introduction:**

A systems approach to understanding late-onset depression points to its association with cardiovascular disease, cerebrovascular disease, tissue and organ trophic disorders, cognitive impairment, anergy, and decreased lifespan. These features are consistent with progressive aging

**Objectives:**

literature analysis

**Methods:**

general scientific method

**Results:**

**Cellular senescence** is characterized by irreversible cell growth arrest. Characteristic features of senescent cells include increased cell size, accumulation of β-galactosidase and lipofuscin in the cytoplasm, accumulation of DNA damage foci, condensed heterochromatin regions, shortened telomeres, and increased expression of cell cycle regulatory markers. Telomere attrition has been linked to depression. Individuals with MDD have shorter white blood cell length compared to subjects without a history of depression. Telomere attrition is also associated with more unstable IL-6 levels and oxidative stress markers in major depressive disorder. Postmortem studies of the brain have revealed that severe depression leads to depletion of various areas of the cerebral cortex and oligodendrocytes. According to the authors, there is a link between severe depressive disorders and cellular aging of the brain. **Mitochondrial dysfunction.** Mitochondrial dysfunction in depression and aging includes genomic instability, defects in biogenesis, electron transport chain and mitochondrial scavenging, nutrient regulation mechanisms, proteostasis. In late depression, the number of mitochondrial DNA copies decreases. Increased production of ROS is a marker of mitochondrial dysfunction. In contrast, depressed individuals have elevated lipid peroxidation markers and an imbalance between oxidative stress and antioxidant stress markers. MtDNA oxidation and fragmentation are consequences of elevated OS, with high levels of fragmented mtDNA being associated with more severe depressive episodes and also a biomarker of aging and tissue damage. In patients with late-onset depression, ccf mtDNA has been found to correlate with chronic levels of IL-6, a master regulator of inflammation and a major component of SASP.

**Conclusions:**

Total scores assess indicators of mitochondrial dysfunction, telomere length, cognitive tests, and structural changes on MRI in women with late depression higher than in elderly men.

**Disclosure of Interest:**

None Declared

